# Human DNA-binding peptidyl-prolyl cis/trans isomerase Par14 is cell cycle dependently expressed and associates with chromatin *in vivo*

**DOI:** 10.1186/s12858-015-0033-x

**Published:** 2015-02-03

**Authors:** Akuma D Saningong, Peter Bayer

**Affiliations:** Department of Structural and Medicinal Biochemistry, Center of Medical Biotechnology, Universität Duisburg-Essen, Room S03 S01 A35, Universitätsstr. 1-5, 45141 Essen, Germany

**Keywords:** Par14, Non-histone protein, Chromatin, DNA-binding, Transcription

## Abstract

**Background:**

Par14, a member of the parvulin family of peptidyl-prolyl *cis-trans* isomerases that is involved in rRNA processing, microtubule formation and the glucose metabolism and has been suggested to play a role in chromatin remodeling on basis of sequence and structural identities to HMG proteins. Par14 is enriched in the nucleus and binds to double-stranded DNA *in vitro.*

**Results:**

By means of sub-nuclear biochemical fractionations, we demonstrate that cellular Par14 is associated with chromatin 3-fold higher than with the nuclear matrix *in vivo*. Par14 is released from the chromatin fraction after treatment with DNase I and elutes at high NaCl concentrations from the nucleic acid-binding fraction. Using qRT-PCR and western blotting we demonstrate that Par14 is up-regulated during the S and G2/M phases in synchronised human foreskin fibroblasts cells.

**Conclusion:**

In the light of our results, Par14 can be described as an endogenous non-histone chromatin protein, which binds DNA *in vivo*. We propose that Par14 is involved in a DNA-dependent activity such as transcription.

## Background

The primary catalytic function of PPIases (EC 5.2.1.8) is to accelerate *cis-trans* isomerization of Xaa-Pro peptide bonds within polypeptide chains [1]. Hence, a role for peptidyl-prolyl cis/trans isomerases (PPIases) in the folding of newly synthesized proteins was inferred and chaperone-like activity has been associated with several PPIases [2,3]. There are few classes among the PPIase family such as the cyclophilins (Cyps) [4], the FK506-binding proteins (FKBPs) [5] and the prototyping Protein phosphatase 2A (PP2A) phosphatase activator (PTPA) [6,7]. Par14 and its isoform Par17 belong to a class of PPIases called parvulins.

Both proteins are encoded by the gene PIN4 which is located on chromosome Xq13.1 within the human genome [8]. The PIN4 promoter is TATA-less and situated in a CpG island typical of housekeeping genes. Human Par14 has a molecular weight of 14 kDa [9] and consists of 131 amino acids. It was first cloned as a homologue of human Pin1 and *E. coli* Par10 [10]. Par14 has an N-terminal basic region (aa 1–35) and a C-terminal PPIase domain (aa 36 – 131) [11,12]. Par17 is an elongated isoform of Par14 that resulted by alternative transcription initiation [8,13]. Whereas Par14 is already present in metazoans, Par17 is only expressed in cells of great apes, but not in those of other primates [14]. In mammalian cells, the expression of Par14 was up-regulated in heart and skeletal muscles [9,10].

Functional studies have shown that both Par14 and Par17 are capable of accelerating the build-up of microtubules [15]. Studies in HepG2 cells and mouse liver revealed that Par14 associates with insulin receptor substrate 1 (IRS-1) via its N-terminal residues, thereby enhancing insulin-induced IRS-1 phosphorylation and affecting glucose metabolism [16]. Immunocytochemistry studies using a Par14 antibody have shown that endogenous protein is accumulated in the nucleoli of cells associating with pre-ribosomal complexes [17]. From cellular fractionation and mutational studies, Par14 showed an uneven distribution pattern between the cytosol and the nucleus fraction [18], and was 2-fold higher in the nucleus than in the cytoplasm.

Based on homologies and similarities of Par14 to members of the High Mobility Group (HMG) of proteins [10] and with structural features within the catalytic domain similar to the transcription factor Lef-1 [14,18], double-stranded DNA constructs were developed and tested for their Par14 binding affinity in fluorescence titration, DNA-cellulose binding and electro-mobility shift assays. Par14 bound preferentially in a 100–400 nM range to bent AT-rich DNA octamers *in vitro*. Such bent AT-rich segments of DNA are supposed to dictate nucleosome positioning [19] and play a role in transcription initiation. Experiments with truncated Par14 showed that the unstructured basic N-terminal part with sequence similarity to the chromatin-unfolding domain of HMGN proteins was indispensable for high affinity DNA binding. Subsequent experiments demonstrated that the phosphorylation of Par14 on Ser19 regulated its sub-cellular localization to the nucleus and its ability to bind DNA *in vitro* [20]. Mutation of Ser19 to Ala abolishes phosphorylation and alters the sub-cellular localization of Par14 from predominantly nuclear to significantly cytoplasmic. Immunostaining showed that a Glu19 mutant of Par14, which mimics the phosphorylated state of Ser19, does not penetrate into the nucleoplasm. As opposed to wild-type Par14, the *in vitro* DNA binding affinity of the Glu19 mutant was strongly reduced, indicating that only the de-phosphorylated protein is active in DNA-binding in the cellular nucleus.

A considerable body of evidence supported the fact that Par14 is a nuclear protein and binds to double stranded DNA *in vitro.* In addition Par14 was reported in immunofluorescence studies to accumulate around chromosomes during mitosis [17]. Moreover, Pin1, a paralogous protein to Par14, is a nuclear PPIase and it has been reported to be involved in the regulation of the cell cycle. In the light of these evidences, it was of paramount importance to investigate the sub-nuclear localization of Par14 by means of biochemical fractionation and to investigate its regulation within the cell cycle making use of cell cycle synchronization deprivation and qRT-PCR.

## Results

### Par14 is a non-histone chromatin protein

**Figure 1:**
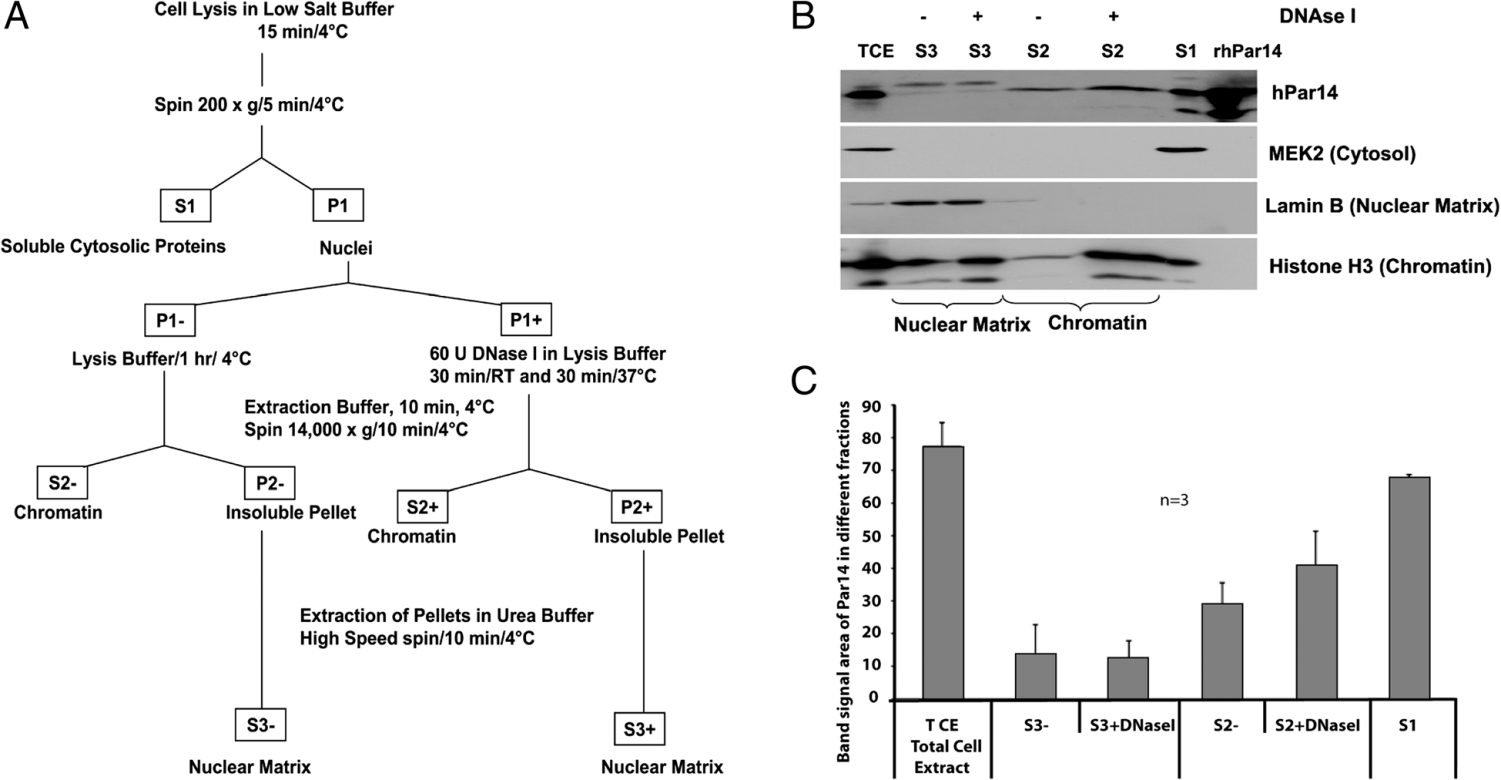
**Par14 localizes more to chromatin than to nuclear matrix. A)** An asynchronous culture of HeLa cells was subjected to biochemical fractionation. **B)** Immunoblotting with the affinity-purified αPPIase, αMEK2, αLamin B1 and αHistone H3 antisera to benchmark the different fractions at a 1:1000 dilution. **C)** Distribution of Par14 in the various fractions quantified by densitometric analysis. The area of the band signals from three independent experiments were measured, normalized and the background signals were subtracted. Multi Gauge densitometry software version 3.1 was used.

A small-scale biochemical fractionation procedure of an asynchronous culture of HeLa cells was performed (Figure 1A). Subsequently, soluble cytosolic proteins (S1), chromatin proteins with and without DNAseI (S2+/S2-) and nuclear matrix proteins with and without DNAseI (S3+/S3-) were isolated. Together with total cell extract (TCE) and recombinant Par14 (rhPar14) these fractions were run on a SDS-PAGE gel, which was further analyzed by western blotting and densitometry (Figure 1B and C). The band area signals for Par14 in the chromatin fractions S2+ and S2- were 3- and 2-fold higher, respectively, than those of the nuclear matrix fractions S3+ and S3-. The densitogram shows the distribution of Par14 in the various fractions (Figure 1C). From the Par14 band signal in S2+ (Figure 1B) it is deduced that Par14 was 1.4-fold released from chromatin when treated with DNase I as opposed to S2-, which did not undergo any nuclease treatment. Hence, there is an enrichment of Par14 in the chromatin fractions when compared to the nuclear matrix fractions. Therefore, the differential release of Par14 from chromatin after DNase I treatment, lends evidence to its intimate association with DNA.

### Par14 binds to DNA *in vivo*

**Figure 2:**
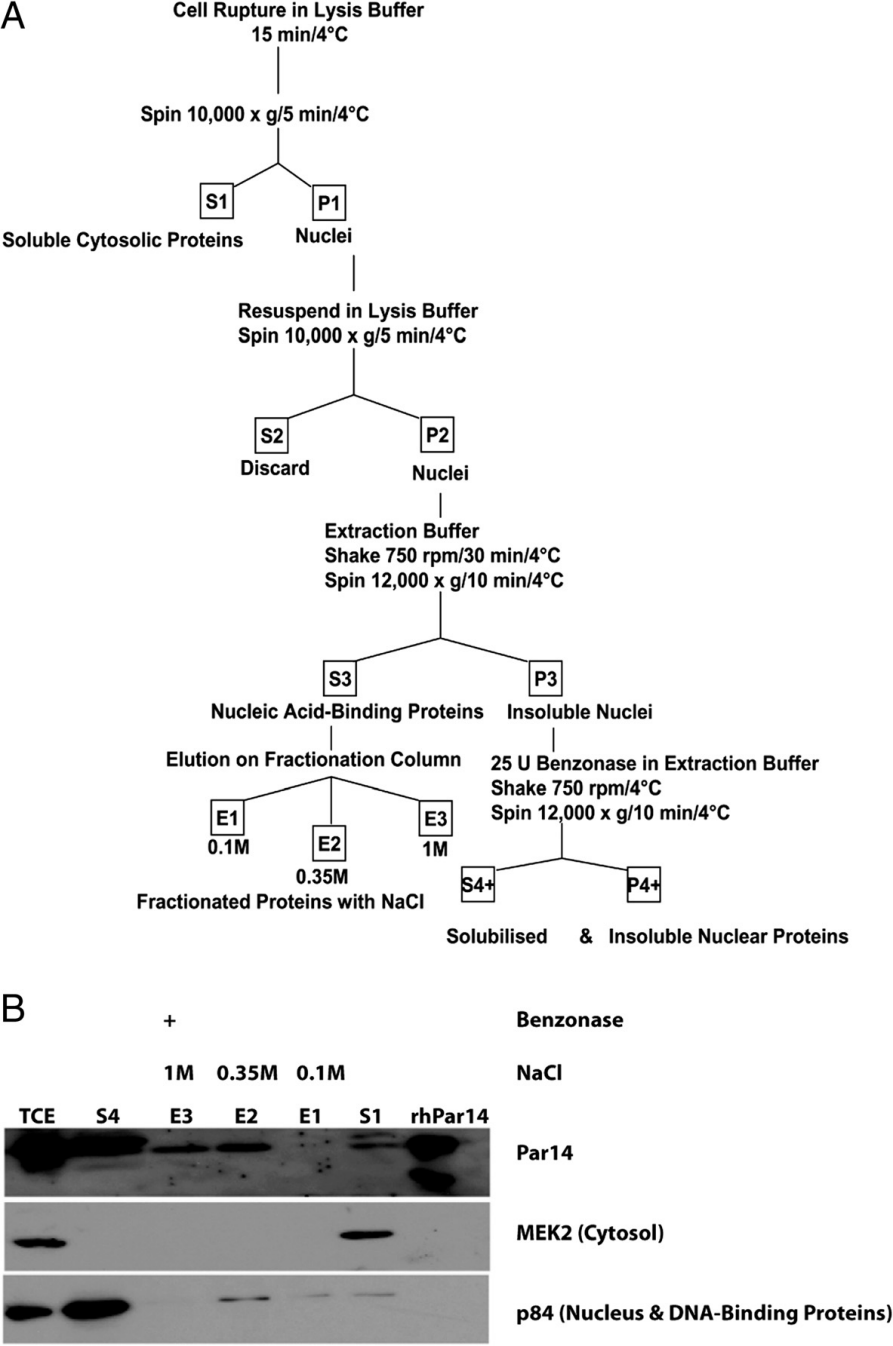
**Par14 binds to DNA** ***in vivo*** **and elutes with increasing salt concentrations. A)** An asynchronous culture of HeLa cells was subjected to a biochemical fractionation of nuclear and nucleic acid binding proteins. **B)** Immunoblotting with the affinity-purified αPPIase, MEK2, and nuclear matrix protein p84 antisera to benchmark the different fractions at 1:1000 dilution.

In concert with our observation that Par14 associates with chromatin and is released after treatment with DNase I, it was mandatory to verify the *in vivo* binding of Par14 to DNA using a different biochemical fractionation approach. Qproteome nuclear subfractionation kit from Qiagen (Germany) was used whereby nuclear proteins are separated to allow for the detection of low-abundance proteins such as transcription factors. Fractions of cytosolic proteins (S1), nucleic-acid binding proteins (E1-E3) and benzonase solubilized nuclei (S4+) were isolated. Eluates were collected using increasing sodium salt concentrations (0.1, 0.35, and 1 M). Fraction S3 contained nucleic acid-binding proteins (Figure 2A) that was later bound on a phospho-cellulose column. Fractions S1, E1 - E3 eluted with 0.1, 035 and 1 M NaCl), S4+ together with TCE and rhPar14 were run on a SDS-PAGE gel. Analysis of the eluates on an immunoblot showed that at 0.35 M (E2) and 1 M (E3) Par14 was eluted (Figure 2B). This finding demonstrated that Par14 was enriched in the fraction that was known as nucleic acid-binding. Thus, Par14 was bound to DNA when isolated from an endogenous cellular environment. S4+ was the supernatant of the nuclei pellet P4+ that was treated with benzonase, and it was referred to as the solubilized nuclei fraction. The presence of Par14 in S1 (cytosol) and S4+ (solubilized nuclei) went to validate previously published data that assigned Par14 to these compartments [20].

### Par14 and polytene chromosomes

**Figure 3:**
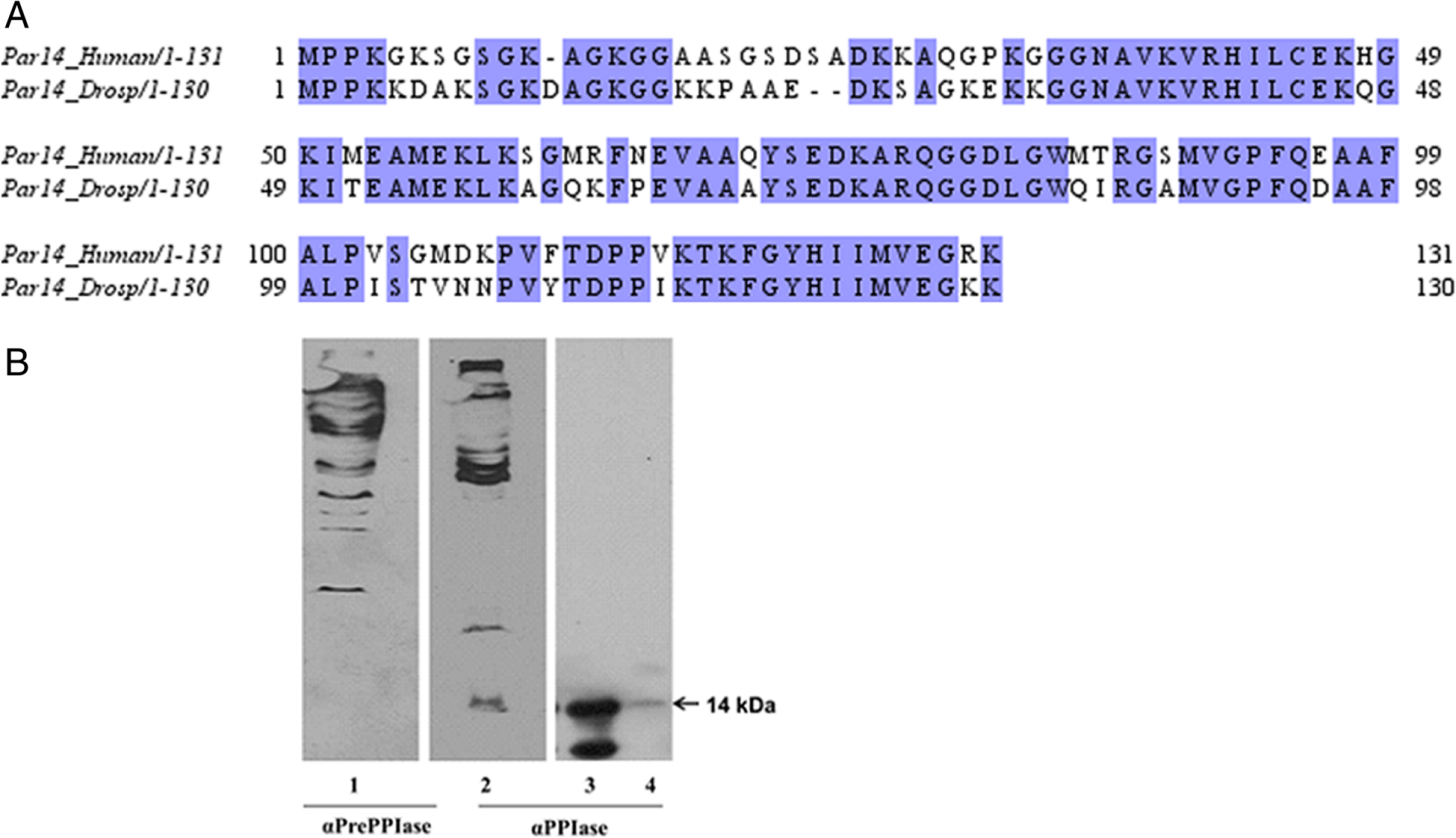
**Par14 is found on the nuclear extracts of** ***Drosophila melanogaster*** **. A)** Sequence alignment of human Par14 with that of *Drosophila melanogaster*. Identical residues are shown in blue. Multiple sequence alignment with the Clustal series of programs was used **B)** Immunoblots of nuclear extracts from *Drosophila melanogaster* Kc cells, recombinant Par14 (rhPar14) and Total Cell Extract (TCE). Lanes 1 and 2: nuclear extract from *Drosophila melanogaster* Kc cells; Lanes 3 and 4: rhPar14 and TCE. A band of 14 kDa is observed on lanes 2, 3 and 4 and not on lane 1 that corresponded to the molecular weight of Par14.

We have shown with ample credibility that Par14 is a chromatin and non-histone protein, which binds to DNA *in vivo*. Our next approach to validate this finding was to investigate if Par14 in Drosophila melanogaster is associated with chromosomes. Human Par14 (CCDS14417) shares 72.5% sequence identity with its ortholog in Drosophila melanogaster (CG11858-RA) and within their PPIase domains, they share 81% sequence identity (Figure 3A). Based on this fact, western blots were carried out using the nuclear extracts (lanes 1 and 2) of Kc cells from Drosophila melanogaster. A protein band of 14 kDa was detected when the αPPIase was used and this band was absent when the pre-immune antiserum of αPPIase was applied (Figure 3B), proofing that the Par14 ortholog is associated with polytene chromosomes. As we have now shown that hPar14 binds to condensed and non-condensed DNA *in vitro* and Par14 is phosphorylated by the casein kinase 2, a serine/threonine-directed kinase implicated in cell cycle control [20], we wondered whether hPar14 might undergo cell cycle correlated expression levels.

### Par14 is up-regulated during the S and G2/M phases of the cell cycle

**Figure 4:**
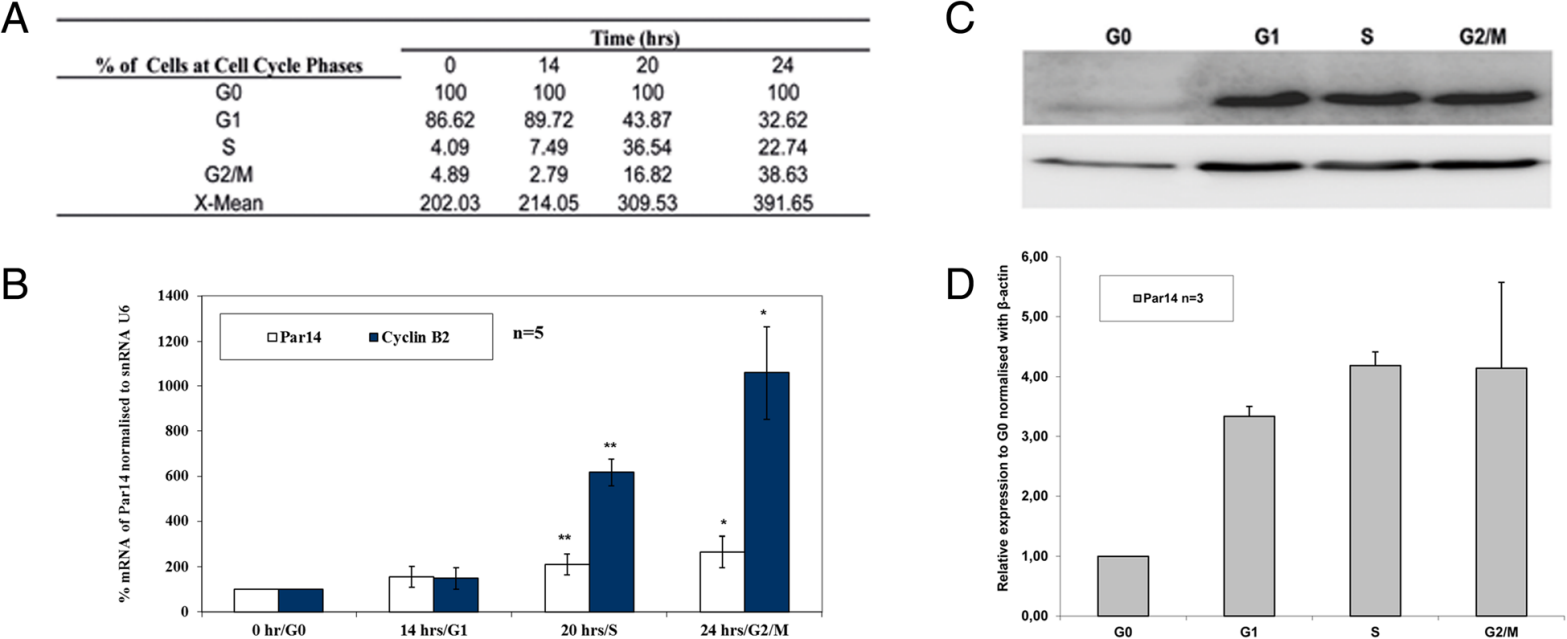
**Up-regulation of Par14 in the S and G2/M phases. A)** Percentage cell cycle distribution. Human foreskin fibroblasts (HFF) were examined by DNA staining with propidium iodide followed by FACS analysis. Numbers denote percentage of cells in G0, G1, S and G2/M phase. Percentage of cells at G0 was fixed to 100 after serum deprivation. The arithmetic mean (X-mean), is an indicator of the fluorescence intensity i.e. the number of propidium iodide molecules bound to DNA. A value above 200 is considered as appropriate. The results show the average of five independent cell cycle synchronisation experiments. For each sample, 1 × 10^4^ cells were analysed **B)** qRT-PCR analysis of Par14 across the cell cycle with synchronised HFF. White columns represent the percentage mRNA expression, dark blue columns the percentage mRNA expression of cyclin B2. After 30–36 hrs of serum deprivation, cells in G0 were arrested at t = 0 h. HFF cells were re-stimulated with 20% FCS. G1 cells were harvested after t = 14 h, S phase cells after t = 20 h and G2/M cells after t = 24 hrs. mRNA levels of Par14 were normalised using snRNA U6. One-way ANOVA was performed using R. The bars represent the standard deviation from five independent cell cycle synchronisation and qRT-PCR experiments conducted. The asterisks on the bars indicate the statistical significance expressed in p-values (** = p ≤ 0.01; * = p ≤ 0.05). **C)** Translational up-regulation of protein across the cell cycle. Western blot analysis was done using αPPIase (1:1000) and β- actin (1:5000) antisera. 30 μg of protein for each cell lysate from the respective phase of the cell cycle was loaded. **D)** Densitometric analysis from three independent cell cycle synchronisation and western blot experiments of Par14 protein. Band signals were normalised with β- actin.

The transcriptional regulation of Par14 across the cell cycle was studied with the aid of qRT-PCR technology. Human foreskin fibroblasts (HFF) were suitable for cell cycle synchronization by serum deprivation. After 30–36 hrs of starvation by depriving cultured HFFs from FCS, an aliquot of cells was obtained and termed G0 cells at t = 0. The rest of the cells were re-stimulated to enter the cell cycle. Aliquots of cells were collected within 14, 20, and 24 hrs after growth re-stimulation for G1, S and G2/M cells respectively [21]. Cell cycle phases were determined by DNA staining and fluorescence-activated cell sorting (FACS) analysis. The distribution of the cells relative to their location during the cell cycle can be seen Figure 4A. Quantitative RT-PCR analyses were done (Figure 4B) using primers for Par14 as previously described [8]. U6 splisosomal RNA (U6 snRNA) was used as an internal control [22] and transcription of cyclin B2 served as a marker gene for the G2/M phase [23]. Transcriptional expression profiles for Par14 and cyclin B2 were normalized against the expression profile for snRNA U6. These results showed that Par14 is slightly but significantly up-regulated 2- and 3-fold in the S and G2/M phases of the cell cycle, respectively, where DNA duplication and cell division takes place. To examine, whether the observed transcriptional cell cycle dependence of hPar14 is reflected on the protein level, we additionally performed western blot studies followed by subsequent densitometric analysis. Par14 is translationally up-regulated within the G1, S and G2/M phases of the cell cycle (Figure 4C) with respect to the G0 phase. The protein shows a ~3-fold increase in G1 and ~4-fold increase in both S and G2/M phases, respectively (Figure 4D).

## Discussion

It has been shown that Par14 is enriched in the nucleus and that it has sequence and structural identities to HMGB and HMGN proteins. The DNA and chromatin association demonstrated in this study appear to be even more plausible when combining these findings with the functions of a series of recently discovered DNA-associated Parvulin binding partners. ASCC2, a member of a novel transcription co-activator complex [24,13], was reported to interact with PIN4 in a yeast two-hybrid screening [25]. In the same assay, polycomb group (PcG) protein EZH2 (Enhancer of Zeste homologue 2), a histone methyltransferase associated with transcriptional repression, was reported as an interactor to PIN4. Fujiyama and colleagues demonstrated that nucleolin interacted with Par14 [17,26]. In co-immunoprecipitation experiments, Rickards and co-authors confirmed nucleolin to be associated with chromatin-containing rRNA genes transcribed by RNA polymerase I only [27]. They proposed that the function of nucleolin is to permit the transcription of nucleolar chromatin by RNA polymerase I. Furthermore, nucleolin was reported as a nuclear protein with a histone chaperone function [28]. Its histone chaperone activity enhanced the activity of chromatin remodeling machineries such as SWI/SNF and ACF. With these several lines of evidence shown, we postulate that Par14 is involved in transcriptional regulation and/or chromatin remodeling based on its association to chromatin.

Beside Par14 other proline isomerases have also been shown to be associated with chromatin. The nuclear FKBP, SpFkbp39p from *Schizosaccharomyces pombe* was reported to be a histone chaperone regulating rDNA silencing and it influenced chromatin organization both *in vivo* and *in vitro* [29]. Still in yeast, Fpr4, a member of the FKBPs in *Saccharomyces cerevisiae* was shown to bind the amino-terminal tail of histones H3 and H4 and catalyzes the isomerization of histone H3 proline P30 and P38 *in vitro* [30]. In the same work, they also showed that the abrogation of Fpr4 catalytic activity *in vivo* resulted in increasing levels of H3K36 methylation and delayed transcriptional induction kinetics of yeast-specific genes. The work summarizes proline isomerization as a novel non-covalent histone modification that regulates transcription and provides evidence for crosstalk between histone lysine methylation and proline isomerization. Above all, Pin1 and its ortholog Ess1, have been demonstrated to modulate the C-terminal domain (CTD) of RNA polymerase II (Pol II) during the transcriptional cycle [31,32]. The CTD is the largest subunit of Pol II and contains repeatedly the heptad sequence (Tyr1-Ser2-Pro3-Thr4-Ser5-Pro6-Ser7) that plays a key role in the transcription cycle, coordinating the exchange of transcription and RNA processing factors. CTD exhibits several structurally flexible heptapeptide repeats that undergo conformational changes in the advent of serine phosphorylation and proline isomerization by Pin1/Ess1, which then have an effect on the transcription of genes. The up-regulation of Par14 in the S and G2/M Phases of the cell cycle in concert with its DNA and chromatin association might be a hint that Par14 also acts on the transcriptional regulation of genes.

## Conclusion

To obtain valuable clues for its cellular function it was of paramount importance to investigate the sub-nuclear localization of Par14. The performed analysis of the nuclear fraction separating chromatin and nuclear matrix proteins ascertains that Par14 is a non-histone protein associated with chromatin. Previous studies from DNA-cellulose binding assays and EMSA have demonstrated that Par14 binds to DNA *in vitro*. Our results from the fractionation of nuclear and nucleic acid-binding proteins strengthen the contention that Par14 binds to DNA *in vivo*. In addition Par14 was reported in immunofluorescence studies to accumulate around chromosomes during mitosis. We have now demonstrated that Par14’s ortholog in *Drosophila melanogaster* is associated with the polytene chromosomes and that hPar14 can be collected from a chromatin protein fraction in human cells. The transcriptional and translational up-regulation of Par14 in the S and G2/M phases of the cell cycle and the dependence of its DNA binding properties on the phosphorylation by the casein kinase 2 relates the action of Par14 to cell cycle regulation and/or DNA repair. In concert with its DNA and chromatin association and the homologies of structural motifs of Par14 to those of the transcription factor Lef-1 these findings suggest that Par14 parallels in part the cellular function of its paralog Pin1, but is acting on non-phosphorylated epitopes of its target proteins.

## Methods

### RNA extraction and RT-PCR analysis

Extraction of total RNA from human foreskin fibroblasts (HFF) cells was performed with TRIzol Reagent (Invitrogen, Germany) according to the supplier’s instructions. Real-time RT-PCR mRNA-quantification was done with the LightCycler system (Roche, Germany). Primers for Par14 5′ to 3′ were: 253-TGG GAG TGA CAG TGC TGA CAA and 254- CAT GTT TTT CAC ATA GAA TGT GTC TGA C [8]. Expression of U6 snRNA (GenBank NR_004394) was used as an internal control constant during the cell cycle with the following primers: forward: CTC GCT TCG GCA GCA CA and reverse: AAC GCT TCA CGA ATT TGC GT. As a positive control, expression of the cell cycle regulating protein Cyclin B2-gene was measured [23]. Cyclin B2-mRNA was detected with the following primers: forward: AAA GTT GGC TCC AAA GGG TCC TT, reverse: GAA ACT GGC TGA ACC TGT AAA AAT. Relative expression changes were calculated using the 2^-ΔΔCT^ method [33,34]. All PCR-products used in the LightCycler mRNA quantifications were confirmed by sequencing to check their identity.

### Eukaryotic cell culture

HeLa cells (DSMZ, Germany) were cultured in growth medium (D-MEM, 1% MEM, 10% heat-inactivated fetal calf serum and 1% penicillin/streptomycin; Gibco, Germany) in T-75 cm^2^ culture flasks (Greiner, Frickenhausen) and incubated at 37°C in a 5% CO_2_ incubator. HFF cells were also grown in the same culture medium like HeLa cells but devoid of 1% MEM. Cells were allowed to grow to 80 - 90% confluence and were sub-cultured as follows: culture medium was discarded and cells were washed briefly two times with pre-warmed PBS, pH 7.4. Thereafter, cells were trypsinized with 1 ml Tryple Express per flask (Gibco, Germany) and incubated for 3 min at 37°C and 5% CO_2_. Cell detachment from the culture flasks with Tryple Express was halted by adding 10 ml of culture medium and the cell suspension was transferred to a 15 ml centrifuge tube. Centrifugation was done for 5 min at 200 × g and the cell pellet was re-suspended in 3–5 ml culture medium.

### Cell cycle synchronisation

HFF cultures were synchronized by serum deprivation. Cells were arrested in the G0 phase before re-entry into the cell cycle [35,36]. 1.5 × 10^6^ HFFs were seeded and allowed to grow to about 65% confluence in culture medium (D-MEM 10% foetal calf serum and 1% Pen/Strep; Gibco, Germany) in a T-300 cm^2^ culture flask (TPP, Germany) at 37°C and 5% CO_2_. The medium was aspirated and the cells were thoroughly washed 3 times with pre-warmed PBS, pH 7.4 to remove any trace of serum. After 30–36 hrs of starvation by depriving cultured HFFs from FCS. The rest of the cells were further cultured in medium containing 20% FCS to re-stimulate them to enter the cell cycle. Aliquots of 1.3 × 10^7^ cells were collected within 14, 20, and 24 hrs after growth re-stimulation for G1, S and G2/M cells respectively [21]. No cell cycle synchronization method is known to date, which achieves a 100% distribution of cells into the respective phases.

### Fixation

Cells gained from the different phases of the cell cycle were permeabilized for intracellular staining. 1 × 10^5^ HFF cells from each phase of the cell cycle were re-suspended in 200 μl PBS, pH 7.4. 200 μl of ice-cold 70% ethanol was added drop wise to the cells under gentle agitation (750 rpm) in a thermormixer and incubated for 15 – 60 min at 4°C. The cells were pelleted at 750 × g for 4 min at RT. Respective pellets were washed with PBS; pH 7.4 supplemented with 1% fetal bovine serum (FBS) and allowed to equilibrate for 20 min at RT while rocking on a shaker. Cells were centrifuged in a swing bucket centrifuge at 1800 × g for 5 min. As a technical hint, a fixed-radius centrifuge will not pellet the cells at this step even at high centrifugal forces. The pelleted cells were ready for use or were stored at −20°C until use.

### Propidium iodide staining and flow cytometry analyses

Before staining with propidium iodide, aliquots of the fixed cells were washed once with PBS, pH 7.4 and re-suspended in 750 μl PBS. After treatment of the cells with 10 μl of 50 μg/ml RNase (Sigma-Aldrich, Germany) for 30 min under gentle agitation with a thermomixer at 37°C. 15 μl of 50 μg/ml propidium iodide (Roth, Germany) were added to the cells and incubated for 5 min making them ready for fluorescence activated cell sorting (FACS) analysis. FACSCalibur flow cytometer (Becton Dickson Science, Germany) was used. Data processing was done with the software CellQuest™ Pro (Becton Dickson Science, Germany). For each sample, 1 × 10^4^ cells were analyzed.

### Production and affinity purification of anti-serum against the PPIase domain of Par14 (αPPIase)

To generate αPPIase polyclonal antibodies, amino acids 36–131 corresponding to the PPIase domain of full length Par14 was cloned into the expression vector pET-41 according to standard procedures, which has been modified with PreScission protease restriction sites [37]. For protein production, the plasmid was over-expressed in *Escherichia coli* strain BL21 (DE3) and purified as a glutathione S-transferase (GST) fusion protein. A total of 2 mg of the recombinant PPIase was sent for immunization of a rabbit (Eurogenetec, Belgium) and western blots were performed to analyse the test bleeds. The final antiserum αPPIase was purified by affinity chromatography using a CNBr-activated Sepharose 4 fast Flow (GE Healthcare, Germany). The purified antibody was used for all western blots conducted to detect both endo- and exogenous Par14.

### Sub-nuclear fractionation

Procedures for solubilization and sequential small-scale biochemical sub-nuclear fractionation were adapted from the method of [38]. In summary, 4 × 10^7^ HeLa cells were harvested by using a cell scraper and spun at 200 × g for 2 min and the supernatant was discarded. The pellet was washed two times with PBS, pH 7.4 and spun down at 200 × g for 2 min. The re-suspension and sulubilization of the pellet was done in 5 ml low salt lysis buffer (10 mM HEPES, pH 7.4, 10 mM KCl, 0.1% Triton X-100) supplemented with a cocktail of protease inhibitors (Roche Diagnostics, Germany) and 1 mM PMSF for 15 min at 4°C. The solubilized nuclei pellet (P1) was recovered at 200 × g, 5 min at 4°C and the supernatant collected was termed the cytosolic fraction, S1 (Figure 2A). P1 was washed again three times with low salt lysis buffer to remove any cytoplasmic contaminants and centrifugation was done like above. The nuclei pellet was re-suspended in 800 μl lysis buffer and separated in two equal volumes of 400 μl and termed P1- and P1+ respectively. Incubation of P1- on ice was done for 30 min. At the same time, P1+ was treated with 30 U DNase I (RNase free, New England BioLabs, Germany) and incubated for 15 min at RT and later for an extra 15 min at 37°C. DNase I in P1+ was inactivated for 10 min at 75°C. Chromatin proteins were extracted from P1- and P1+ by adding 500 μl of extraction buffer (1% Triton X-100, 50 mM HEPES, pH 7.4; 150 mM NaCl, 30 mM Na_4_P_2_O_7_ · 10 H_2_O, 10 mM NaF, 1 mM EDTA) containing a cocktail of protease inhibitors (Roche Diagnostics, Germany) and 1 mM PMSF, for 15 min at 4°C respectively. Both P1-/P1+ were centrifuged at 14,000 × g for 10 min at 4°C and the respective supernatants S2-/S2+ were collected and named chromatin fractions. The resulting pellets P2-/P2+ were solubilized in 250 μl urea buffer (8 M urea, 100 mM NaH_2_PO_4_, and 10 mM Tris–HCl, pH 8.0) and clarified by centrifugation at high-speed in a micro-centrifuge for 10 min at 4°C. The supernatants S3- and S3+ collected respectively were named nuclear matrix fractions.

### Fractionation of nuclear and nucleic acid binding proteins

Qproteome nuclear subfractionation kit (Qiagen, Germany) was used for the fractionation procedure and the extraction of nucleic acid binding proteins. The manufacturer’s protocol was used and the scheme of the procedure is illustrated in (Figure 3A). A phosphor-cellulose column provided in the kit was used for the elution of nucleic acid binding proteins. The eluates E1, E2 and E3 were concentrated and desalted by chloroform/methanol precipitation [39]. Bradford assay was used to determine the concentrations of S1, E1, E2, E3 and S4+ and equal amount of protein from the fractions was loaded for electrophoresis and subsequent western blotting analyses.

### SDS-PAGE and immunoblotting

SDS-PAGE and immunoblotting were carried out according to standard protocols with the following notations. After determining the protein concentrations by the Bradford method, 30 μg protein per lane except otherwise stated were resolved on 12.5 or 15% SDS-PAGE gels [40]. Electrophoretic separation was carried out for about 2 hrs at 125 V in a Novex MiniCell Chamber (Invitrogen, Germany). Blotting was done on a nitrocellulose membrane (Invitrogen, Germany) in transfer buffer (25 mM Tris–HCl, pH 8.0 – 8.3, 192 mM glycine, 20% methanol) at 30 V for 1 hr using the semi-dry Fastblot apparatus (Biometra, Germany). Immunoreactions on the membranes were visualized with the use of an enhanced chemiluminescence (ECL western blot reagents) kit followed by exposure on CL-XPosure Film (Thermo Scientific, Germany). Protein bands on the film were developed using the AGFA Curix 60 developer (AGFA, Germany).

### Determination of Par14 protein expression

Total Cell Lysates (TCE) were obtained from HFF cultures synchronized by serum deprivation and processed for immunoblotting as described above. Normalisation of Par14 expression was done with β-actin. Firstly, the expression of β-actin at G0 was obtained by dividing the value of its band signal area by its self and a factor of 1 was obtained. This was done because we considered cells at G0 to be resting and thus no relative expression changes. Secondly, the relative expression of β-actin at G1, S and G2/M phases was calculated with respect to its expression at G0. This was done by dividing the values of the respective band signal areas at G1, S and G2/M by the value of the band signal area at G0. This can be explained by the following equation for the relative expression at G1:

Relative Expression of β-actin at G1 = (Band signal area at G1 – Background signal)/(Band signal area at G0 – Background signal)

Normalisation of Par14 expression in the various phases of the cell cycle was derived by dividing the value of the band signal area of Par14 at the phase in question by the relative expression of β-actin during this phase:

Normalisation of Par14 at G1 = (Band signal area of Par14 at G1- Background signal)/(Relative expression of β-actin at G1)

Fold changes of Par14 at G1, S and G2/M were done by dividing the normalised value of Par14 at the respective phase by its normalised value at G0:

Relative expression of Par14 at G1 = (Normalised value of Par14 at G1)/(Normalised value of Par14 at G0)

Calculations for the S and G2/M phases were done in the same manner. The results in Figure 4D were obtained from three independent and averaged experiments.

## Abbreviations

EDTA: Ethylene-diamine-tetraacetic acid
FACS: Fluorescence-activated cell sorting
FBS: Fetal bovine serum
FCS: Fetal calf serum
HFF: Human foreskin fibroblast cells
Par14: Parvulin 14
Pin1: Peptidyl-prolyl cis-trans isomerase NIMA-interacting 1
PMSF: Phenylmethylsulfonyl fluoride
PPIase: Peptidyl-prolyl cis/trans isomerase
